# Palliative care as a digital working world (PALLADiUM) - A mixed-method research protocol

**DOI:** 10.1186/s12904-023-01173-w

**Published:** 2023-07-22

**Authors:** Sandra Grimminger, Maria Heckel, Moritz Markgraf, Sarah Peuten, Moritz Wöhl, Henner Gimpel, Carsten Klein, Christoph Ostgathe, Tobias Steigleder, Werner Schneider

**Affiliations:** 1 Palliativmedizinische Abteilung, Comprehensive Cancer Center CCC Erlangen-EMN, Universitätsklinikum Erlangen, Friedrich-Alexander-Universität Erlangen-Nürnberg, Krankenhausstraße 12, 91054 Erlangen, Germany; 2 FIM Research Center, University of Augsburg, Universitätsstraße 12, 86159 Augsburg, Germany; 3 Project Group Business & Information Systems Engineering of the Fraunhofer FIT, Universitätsstraße 12, 86159 Augsburg, Germany; 4 Professorship for Sociology, University of Augsburg, Universitätsstraße 10, 86159 Augsburg, Germany; 5 Chair of Digital Management, University of Hohenheim, Schloß Hohenheim 1, 70599 Stuttgart, Germany

**Keywords:** Palliative care, Terminal care, Digital technology, Communication, Multi-professional

## Abstract

**Background:**

In Palliative Care, actors from different professional backgrounds work together and exchange case-specific and expert knowledge and information. Since Palliative Care is traditionally distant from digitalization due to its holistically person-centered approach, there is a lack of suitable concepts enabling digitalization regarding multi-professional team processes. Yet, a digitalised information and collaboration environment geared to the requirements of palliative care and the needs of the members of the multi-professional team might facilitate communication and collaboration processes and improve information and knowledge flows. Taking this chance, the presented three-year project, PALLADiUM, aims to improve the effectiveness of Palliative Care teams by jointly sharing available inter-subjective knowledge and orientation-giving as well as action-guiding practical knowledge. Thus, PALLADiUM will explore the potentials and limitations of digitally supported communication and collaboration solutions.

**Methods:**

PALLADiUM follows an open and iterative mixed methods approach. First, ethnographic methods – participant observations, interviews, and focus groups – aim to explore knowledge and information flow in investigating Palliative Care units as well as the requirements and barriers to digitalization. Second, to extend this body, the analysis of the historical hospital data provides quantitative insights. Condensing all findings results in a to-be work system. Adhering to the work systems transformation method, a technical prototype including artificial intelligence components will enhance the collaborative teamwork in the Palliative Care unit.

**Discussion:**

PALLADiUM aims to deliver decisive new insights into the preconditions, processes, and success factors of the digitalization of a medical working environment as well as communication and collaboration processes in multi-professional teams.

**Trial registration:**

The study was registered prospectively at DRKS (Deutsches Register Klinischer Studien) Registration-ID: DRKS0025356 Date of registration: 03.06.21.

## Background

Digital transformation is more and more present in health care. This digital transformation represents a great opportunity yet also great challenge. It is becoming apparent that even areas that have already been largely transformed are facing major difficulties in implementation and integration. While the advantages and opportunities are rather clear from the technical and the health economic perspective [1], clinics encounter problems in the introduction and application [2]. Additionally, from the sociology of work and organization perspective, there are also reservations when digitalization processes lead to formalization and standardization, which in turn come at the expense of genuine work structures and processes [3].

Overall, digitalization in the health sector presents opportunities and challenges at the same time. Therefore, it requires a multidisciplinary approach including (socio-)technical, medical and social science for a comprehensive insight. Palliative care, for example, is a discipline focusing on holistic care in the last phase of life and does not primarily aim to cure the underlying disease but to minimize suffering (multidimensional: physical, psychological, social, and spiritual) among patients and relatives with special focus on their individual needs [4, 5]. For this purpose, and due to this characteristic multidimensionality in palliative care that requires a high degree of flexibility in the treatment procedure an intensive exchange of information and knowledge occurs regularly in a multi-professional team with actors from different competence profiles (including medicine, nursing, psychology, social work, physiotherapy, music and art therapy, spiritual care, and volunteers). In multi-professional teams, inter-subjective shared information and subjectively available knowledge are used to produce jointly shared, orientation-giving, and action-guiding practical knowledge for the specific case (situational knowledge) and cross-case practice (generalizable knowledge) [6]. However, due to these multi-professional perspectives, close collaboration is of high importance. In a setting such as Palliative Care, which is rather remote from technology and digitalization, there are no common sustainable digital concepts for these communication and collaboration processes so far. Digital technologies, namely desktop computers and laptops, are used to fulfill the minimum criteria regarding documentation. Still, collaboration processes run in parallel despite the importance of the information in the documentation [7, 8]. International initiatives have been formed to standardize the data structure in Palliative Care [9]. This, however, is only a foundation to improve collaboration digitally. Digital improvement of communication has so far been limited to sharing electronic medical records, and even this simple innovation leads to a sustainable improvement of care [8, 10]. Digital transformation research in Germany rarely explicitly addresses Palliative Care [11] and has so far focused primarily on medical and nursing aspects in general [12], healthcare robotics [13, 14], (outpatient) patient care, and the problematization of human-technology interaction [15]. The impact of a digital system that supports communication flows in teams and in which user-type specific interactivity has not yet been investigated in Palliative Care. On the one hand, existing systems in the ward, such as the Hospital Information System (HIS), serve other purposes than collaboration (e.g., billing). On the other hand, available collaboration software (e.g., Microsoft Teams, Slack, or Trello) lacks the specifics of and integration into the medical context. Hence, appropriate, field- and case-sensitive digital support and documentation can promote this process and help structure multi-professional collaboration [16]. PALLADiUM aims, besides the development of a demonstrator, to identify parameters for basic design decisions of digital tools that can be usefully applied in sensitive areas such as palliative health care. Special consideration is given to the non-technical aspects of communication and collaboration, which play a particular role in the field of Palliative Care. The focus is on supporting the communication and collaboration of the multi-professional team (information and knowledge flows), in a profitable way. For this, it is essential that all team members have access to the information that is respectively relevant to them and that they know what the information entails for their own work. Communication with patients and perceived treatment effects from the patients' perspective are not the subject of this study.

In Germany, there are no guidelines or regulations in this regard and no digital application. Although a lot of data is collected in everyday clinical practice, it is not processed for the information demand of specific occupational groups and linked in a way that aims to improve communication and collaboration among employees. There has not yet been any research on digitalizing team-internal communication and collaboration in Palliative Care. Still, it is in line with current trends in Palliative Care [17]. To address this research gap, the three-year project PALLADiUM focuses on investigating communication and collaboration in a Palliative Care unit at a hospital. The research site is the University Hospital Erlangen, a maximum care center with 25 clinics, 56 interdisciplinary centers and more than 9,500 employees, providing more than 1,300 beds. The cornerstone of communication and collaboration in the investigated Palliative Care unit is a weekly multi-professional team meeting (WMPT). The meeting brings together Palliative Care team members from all professional groups (including physicians, nurses, clergy, therapists) to discuss patients. PALLADiUM focuses on this cornerstone, the WMPT, yet also investigates the related upstream and downstream communication and collaboration (e.g., doctor's visits, shift handovers, everyday work processes, etc.). In an interdisciplinary approach, social scientists first observe and investigate communication and collaboration in the Palliative Care unit for the grounded theory approach [18]. The resulting qualitative findings (e.g., with regard to different information needs or information and knowledge gaps) serve as a major input for the involved information systems engineers modeling the communication and collaboration in the Palliative Care unit with respect to the *work systems theory* [19, 20]. Hence, this project adheres to the Work Systems Theory [19, 20] and sticks to the *work systems method* [19, 20] as an overarching methodological approach. In the project, these are applied to the work system of Palliative Care, specifically to communication and collaboration processes, and are expanded to include perspectives and methods from sociology. Besides primarily qualitative methods from sociology, the analysis of historical structured (numeric) and unstructured (free text) data from the HIS, including the application of methods in the area of Artificial Intelligence (AI; particularly natural language processing), aims to develop a technical prototype as a supportive tool. Thus, depending on the professional group, the structured (and automated) processing of relevant information can contribute significantly to ensuring that each professional group has the relevant information available for the treatment in order to carry it out in the best possible way, without the role of existing (experiential) knowledge and cooperative coordination in the team losing importance. Rather, the aim is to show how digital information systems can support and optimize the cooperation and knowledge processes in the team as well as for the individual. The comparatively low progress in digitalization in the Palliative Care unit represents an outstanding opportunity to conduct prototypical research into how information and communication technology and digitalization processes can be designed at an early stage and which working conditions and competencies must be created among the actors involved in patient care to enable acceptance and confident handling of digital technologies. The project aims to deepen existing knowledge and extend the scientific body of knowledge by pursuing the following primary research questions:

- How should a digitally-enabled Palliative Care work system be designed to be feasible and accepted by the team members?
- Which AI-based approaches are suitable for making structured and unstructured data in Palliative Care more usable for communication and collaboration processes?
- Which digital competencies are required of team members in the work system to make a digital transformation in Palliative Care successful?

## Method

As diverse as the research approach, the project team includes three main professions (i.e., sociology, information systems engineering, and Palliative Care). To explore the questions mentioned above, the project favors a multi-mixed-method research approach [21] applied in an iterative procedure:

The overarching methodological framework is the Work System Method [19, 20], respectively the Work System Transformation Method [22], which we apply particularly to communication and collaboration processes in Palliative Care. The Work System Method has three steps: I) identify the system and its opportunities, II) analyze the system and identify possibilities in it, and III) recommend and justify changes [20, 23]. All three steps are closely interlinked with qualitative data collection and analysis to successfully adapt the work system theory and make it usable for the Palliative Care environment. This includes the exploration of the field (the inpatient Palliative Care unit), which is an essential step to create a deeper understanding of multi-professional communication in the working environment and, thus, provides functional and non-functional requirements for an information system facilitating communication and collaboration. Moreover, this is crucial to identify the work system in an as-is and to-be state. The work system (i.e., its processes and activities, information, and technologies) serves as a blueprint for a technical prototype aiming to improve communication and collaboration in the multi-professional team of the Palliative Care unit. The Palliative Care unit of the University Hospital Erlangen, affiliated with the Palliative Care research department, was chosen as the sample due to access and proximity. It has six single and three double rooms and treats approximately 350 inpatients each year.

Besides the qualitative research-driven stream, pre-existing data are the basis for the quantitative research-driven stream in this project. Applying methods from the area of AI – i.e., machine learning and natural language processing – provides insights extending the deduced qualitative insights. Furthermore, besides the identified requirements, the historical data serves as the basis for AI models extending the functionalities of the technical prototype—particularly with regards to text processing and preparation.

Regarding technical development, the project favors an iterative approach with early on-field phases to reevaluate the progress and reconsider design decisions. As early as after 18 months, the first version of the prototype shall be ready for on-field tests. This will serve as a basis for further development of the work system and the functional prototype. The prototype exemplifies the added value for the working environment of Palliative Care and enables an approximate quantification of the added value in exemplary cooperation processes. It will mature continuously in iterative project sprints, using findings from qualitative research. Any need for change or new requirements that become evident through qualitative research will be reflected to improve the design.

## Qualitative methods

**Fig. 1:**
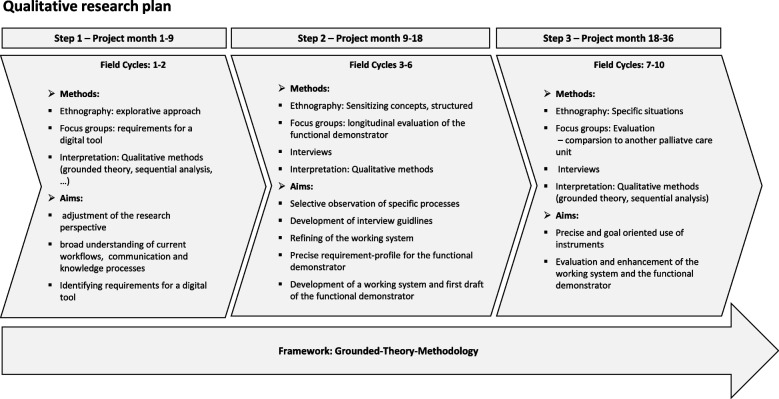
Phases of the qualitative research approach

The focus of the qualitative research approach is to initially map the actual and target state of digitalization and digitalization possibilities of the cooperation processes in the Palliative Care unit. Later on, its focal point will shift to an evaluative orientation. Ethnographic fieldwork centered around the WMPT will provide us with insights about information and knowledge flows in and around the WMTP, communication strategies of individuals and in the team, as well as the usage of analog and digital tools. The ethnographic approach [24, 25] will supply us with an understanding of the participant's tacit knowledge and everyday practices that would not otherwise be accessible. The qualitative data collection and analysis will be carried out according to grounded theory, a structured yet flexible methodology [18]. The main characteristic of grounded theory is the constant alternation between fieldwork (data collection) and data analysis/theory building. Core features are conceptualization (through open, axial, and selective coding procedures), permanent comparison, theoretical sampling, and memo writing [26]. For the recruitment regarding the ethnographic fieldwork (participant observation, interviews, and focus groups), all employees of the ward who regularly work in the team and with patients are included anonymously. For the interviews and focus groups, participants from various professional groups (physicians, nurses, therapists, pastoral care, psychotherapy, as well as social work and case management) were specifically asked to represent as broad a professional spectrum as possible. Participation is voluntary, based on informed consent, and can be taken during working hours. No further compensation or incentives are given. The findings from the research are reflected to the team in focus groups and on-site presentations. Supervisors have access to the results of the surveys only in anonymized or aggregated form (Fig. 1).

### Ethnographic fieldwork

Ethnographic fieldwork ensures that the specific relevance of actor-bound knowledge in the Palliative Care setting is incorporated into the work system and prototype development.

### Participant observations

Ethnographic field studies [27] take place at the Palliative Care unit in the university hospital in Erlangen. Two researchers (SG, SP) with focus on qualitative social research, sociology of death and dying and sociology of knowledge will accompany and observe employees from several professional backgrounds in their daily working routines. They will observe their practices and interactions while taking field notes and memos. Information about the professional background of the researchers, research interests and details about the project are shared with the team members situationally during the ethnographic research. The data will be collected in three steps, each containing two to three field cycles. Every field cycle will last two weeks and is centered around the WMPT, one week before and one week after. The WMPT is a node point in multi-professional collaboration, where expertise and case-related knowledge are knit together. The pauses between the on-site visits are used to interpret the collected data. Distancing and reflection (writing process, exchange within the sociological team, memos) on one's research role, presumptions, experiences, and perceptions in the field and their potential impact on data analysis and further research decisions is also important here [28]. Field notes will be elaborated into protocols, coded in MAXQDA, and analyzed using the grounded theory approach [18]. The resulting hypothesis and communication and collaboration models will be regularly discussed in the interdisciplinary research team and used to create and refine the work system's design and the functional prototype. According to grounded theory, findings will be used to concretize the focus of following ethnographic observations. Since the participant observation is based on the observation of interactions, no ns according to a guideline, such as in an interview, are needed. Conversations arise situationally and are also recorded in the field notes. With each field cycle, the observation is specified. As the empiricism progresses and through the evaluation, the foci for the subsequent observations are successively worked out and refined.

The research follows an explorative design in the first stage (field cycle 1–2). The aim is to get a broad picture of communicative and collaborative processes and progress in the Palliative Care unit. This will provide a fundamental understanding of interdependencies between the different working flows of the multi-professional team and the usage of digital and analog tools, what role they play in daily practices and where they might be deficient. Therefore, practices, patterns, manners, places, and situations in which the Palliative Care staff members make decisions and participate in communicative interaction are observed. Since two researchers are simultaneously in the field and will look at the same processes, different perspectives can be contrasted. The first stage is essential to concretize the research perspective. We also devise the first sensitizing concepts [29] to elaborate and specialize our observation focus for the following field cycles. The second stage (field cycle 3–6 observations will be more structured and centered on explicit research questions. Both researchers will be simultaneously on-site but may follow different objectives in their observation. During this period, field-specific interview guides will be developed. During the third stage (field cycle 7–10), both researchers might (but do not have to) be present independently at the research site. At this stage, highly selective observations will be conducted. By the 18^th^ project month, a first draft of the overall system in the form of a Minimum Viable Product (MVP) will be available and introduced to the Palliative Care unit. Participant observations and focus groups will aim to produce insights about the practical usage, hindrances, and opportunities for prototype enhancements, as well as the acceptance and interactivity in human–machine collaboration.

### Interviews

Semi-structured interviews (SG, SP) will be conducted with the staff. Three to four members of every profession that's part of the patient-involved Palliative Care team (doctors, nurses, therapists and spiritual care, social workers) will be questioned at two or three different points in time based on an informed consent agreement. The first wave of interviews (field cycle 3–4) will focus on the participants' perspectives on their collaboration and communication processes, their usage of digital tools, and their expectations of those. In field cycles 5–10, it is planned to focus the interviews on evaluating and assessing early versions of the digitally assisted work system and its artifacts. Palliative Care.

The design of Interview guides stems from findings from participant observations and respective necessities regarding the work system design (e.g., specification, clarification, evaluation) and will therefore be adjusted to the peculiarities of the field. The specific questions arise from empiricism and are not fixed at the beginning. All Interview transcriptions will be transferred to MAXQDA, coded, and interpreted based on grounded theory.

### Focus groups

Between three and six thematic focus groups [30] with national and international experts on digitalization, hospice, and Palliative Care will be conducted (SG, MM, SP, MW). Those experts will be recruited based on their relevant publications or practical experience.

The first focus group (research cycle one to two) will center around requirements and expectations for a digital working assistance tool. We will ask questions about the information exchange and loss in their everyday work, what information needs there are, and what information, for example, should immediately be forwarded to all team members or employees in certain professional groups. A formative evaluation of the prototype's longitudinal use is planned in subsequent focus groups. At the same time, it serves as a basis for the preparation and scaling of an implementation of the design into a functional working system. The aim is to provide a well-founded analysis of the possibilities and limits of the developed prototype (summative evaluation). In field cycles nine to ten, the results and proposed solutions for the field of investigation at the University Hospital Erlangen are critically reflected at a second location: The Palliative Care unit of the University Hospital Augsburg, to allow for a systematic comparison of the conditions and possibilities for implementing the developed solutions based on empirical findings from research at University Hospital Erlangen. Transfer possibilities will be discussed in focus groups. As a reflection of the results with another inpatient location, a refinement or generalization of the design principles of the digitally supported work system is expected here.

### User typology-specific processing and evaluation of (un)structured data and information

The findings from qualitative research will define the outline for the design of the work system design as well as of the functional prototype. Based on the results of the qualitative research, the project team will identify functionalities to be implemented in a prototype to facilitate the work system. Each functionality will be evaluated based on expected impact (fostering collaboration), feasibility, and development effort. Then, the project team prioritizes the development of the functionalities of the prototype based on the dimensions mentioned above. According to the prioritization, the prototype is iteratively developed until an MVP state is reached and initially evaluated in the field. Based on the insights gained from this, the prioritization of the remaining functionalities may be adjusted, and the further development of the MVP will then again follow the prioritization made by the research team.

The prototype will use quantitative data from the local HIS. For Palliative Care, structured data are usually available in the form of biometric (laboratory data, vital signs) and psychometric data (including symptom assessment, self-reported or recorded by others) [31, 32]. On structured data, very basic concepts like correlation or linear regression are already useful to understand structured data and draw objective conclusions properly. This however is not in scope as the focus is on collaboration support rather than support of the therapy itself.

Additionally, unstructured data are available in image and text form for biometric information (including examination findings from equipment such as Magnetic Response Imaging or tissue samples) as well as for psychometric information (e.g., specialist psychological exploration and interview documentation from various members of the multi-professional team). The progressive development in the field of natural language processing, favoring language models such as BERT [33] or GPT-3 [34], enables the systematic integration of text-based data and retrieving information from them. A known challenge in Palliative Care is the abundance of data (primarily text-based documentation). A possible approach to tackle this challenge is individualized summaries of existing documentation for a patient (abstractive or extractive summarization), question-answering, or identification of triggers for actions, to speed up information retrieval. Modern large language models are zero-shot learners, hence, they can be applied without training, simply by defining corresponding prompts for the given context.

Overall, combining quantitative and qualitative information from various sources and a profession-specific preparation of it enables a more holistic understanding of the patient. To make use of them, a well-balanced information system is required. Hence, the project contributes to computer-supported cooperative work, collaboration engineering, group support systems, collective intelligence, and hybrid (human and artificial) intelligence. Here, the project will provide new insights into how information technology not only supports communication but also how AI-based systems actively support collaboration processes, potentially up to a role as an interaction partner that takes the initiative and autonomous role in the communication and collaboration processes. In concrete terms, an AI-based system can act as an additional actor and provide relevant contributions through comprehensive analytics and connotation-free representation.

In all areas of health care and beyond, the use of unstructured data in multi-professional teams is a challenge. PALLADiUM will explore the AI-based use of unstructured data in the context of the working environment of Palliative Care. Concepts of AI-based clinical decision-making have been researched for two decades [35]; however, this research activity is limited to evaluating scientific data. So far, this innovation has hardly found its way into clinical care. PALLADiUM addresses both the clinical-application-related AI-based use of unstructured data and aspects of human–machine collaboration to integrate this use into clinical workflows in a direct prospective comparison of AI-based and clinical-empirical recommendations for action and thus represents a considerable scientific gain in knowledge.

### Expected results

The central result of the project will be a knowledge-based, evaluated work system design of digitally-enabled and digitally-supported communication and collaboration processes for multi-professional Palliative Care teams to make case-relevant information and knowledge jointly available and usable. In particular, PALLADiUM should contribute to transferring work systems theory to the specifics of work systems in health care, nursing, and present design principles for work systems in this field. PALLADiUM is expected to deliver decisive new insights into the preconditions, processes, and success factors of the digitalization of a medical working environment and communication and collaboration processes in multi-professional teams. The design principles derived from this basic research should be transferable to other healthcare contexts in which multi-professional collaboration is crucial (e.g., inpatient hospices, geriatrics, outpatient Palliative Care).

## Discussion

### Ethical considerations

Within the study, no interventions are expected to cause any unwanted side effects. The study will be explained to all participants, including the purpose and the methods, questions will be clarified, and participation will take part based on signed, written, informed consent. Participants will be informed that if they wish to withdraw from the study, they can do so at any time without giving their reasons. Ethnographic attendance will be oriented on the individual participants' available time and workload peaks. Interviews will take place during the staff's working hours and will be planned according to their schedule. Anonymized historical clinical data from the HIS used to finetune the natural language processing approach and is used under a broad consent agreement. Of course, the project operates under ethical approval, an audited data protection concept, and the staff council representatives' consent.

Even though the ethnographic researchers care not to disturb any processes, their mere presence might be challenging for some staff members. Since the researchers coordinate their attendance in situ and cooperate with the individual members of the Palliative Care team, staff members can always decide about the researcher's participation in every situation. If non-participants (patients, relatives) are present, they will be informed about who the researchers are. For conversations between team members and patients or their relatives, they are asked in advance by the respective team members whether the researchers are allowed to be present or not. The development of digital artifacts to improve multi-professional teamwork should be beneficial for the staff of the digital care unit. Also, the findings on the topics of communication and collaboration will be shared with participants, which will give them feedback on their organization and processes and could improve their awareness of everyday practices. Results from the project may be transferred to other sectors of the healthcare sector.

### Data security

All data will be handled strictly confidential. Field notes, as well as interview transcripts, will be pseudonymized. The document linking names and personal data to the pseudonymization keys is locked in a secure place in the Palliative Care research department facilities in Erlangen. Consent forms will be kept separately from the data. The data is stored at the servers of the University Hospital in Erlangen and secured by password. Because some members of the research team are direct superiors to the staff of the Palliative Care unit (the participants), a data management plan was formulated that prohibits those team members from access to non-pseudonymized data. Therefore, recorded audio files and field notes will be stored in a separate protected space on the Erlangen Universities servers. Only researchers that don't have this affliction know the password and have access to the list that links pseudonyms with the names and data of the participants. After data analysis, the list containing the encryptions will be deleted. No information will be included to identify participants when any research results are published and shared at conferences. Clinical Data from the HIS, will be pseudonymized and safely transferred between the University Hospital of Erlangen (Palliative Care) and the University of Augsburg (business informatics).

### Funding and cooperation

PALLADiUM is funded by bidt (Bavarian Research Institute for Digital Transformation), which is part of the Bavarian Academy of Sciences and Humanities.

The digital transformation of the working world of Palliative Care affects the professional actors to the same extent as the patients and their families. To do justice to this, PALLADiUM envisages the involvement of these stakeholder groups as well as the public. Therefore, PALLADiUM cooperates with an advisory board, which provides professional networking and gives valuable indications and ongoing peer review. PALLADiUM cooperates with the Coordination Office for Hospice and Palliative Care in Germany, funded by the Federal Ministry for Family Affairs, Senior Citizens, Women, and Youth, and with the All Ireland Institute of Hospice and Palliative Care (AIIHPC). To involve the public, patient, and family representatives, the University Hospital Erlangen and the AIIHPC integrate their patient and public involvement initiatives. The cooperation takes place within the framework of twice-yearly events, in which the research questions, solution approaches, and the current project status are discussed. In the sense of active participation, research aspects from the respective project status are derived from the events and integrated into PALLADiUM. The collaborations also provide public scrutiny and support the scientific-ethical integrity of PALLADiUM beyond the project partners' research excellence and integrity.

### Dissemination

Results will be written up and published in peer-reviewed journals. Findings will also be presented at national and international conferences in the fields of Palliative Care, information systems, and sociology. For some publications, anonymized data will be shared in open-access platforms under the FAIR Principles. PALLADiUM is presented on the homepages of the University Hospital Erlangen and the University of Augsburg and made accessible to the interested public and other researchers. It is planned to link these pages with the homepages of the German and international cooperation partners as well as the homepage of the bidt. Research results will also be made available here, including the fully anonymized data and annotated data sets—as far as possible under data protection law—and made available for scientific cooperation. PALLADiUM also publishes a compendium and instructions on the scientific methods used in the project.

Furthermore, the guidelines for developing a tutorial for digitizing multi-professional work environments derived from PALLADiUM are published here. The aim is to enable multi-professional teams (in healthcare) to initiate a process of reflection on their collaboration, to improve collaboration based on the insights gained during this project. The open research approach aims to make the knowledge gained in PALLADiUM and the data collection available for further scientific use and promotes further scientific cooperation on digital transformation. It is planned to organize public events where the research results are presented and discussed in a layman-friendly way. Key results will be communicated in educational workshops. So far, a press release has been published, as well as a short description on the websites of the Palliative Care Department at the University Hospital Erlangen, the University of Augsburg, and the bidt, respectively.

The expected knowledge gain and routines from the research will radiate into the ward at the University Hospital Erlangen due to the close involvement in the project. Bringing the developed prototype into a production setting is not intended as of now. Further, the insights gained during the project are intended to disseminate into other Palliative Care wards and other healthcare areas, with the intended communication outlined above.

### Risks

There is a possibility that it will not be feasible to introduce and install the functional prototype in the Palliative Care unit. This might be due to the interface of the HIS that might not allow for adding new elements to the running system. If this is technically possible, we would still have to be certain that an interruption of running operations is impossible. In addition, the HIS will be exchanged during the research period. Since we want to avoid double documentation that takes extra time and might disturb the routines and the effectiveness of the work done at the Palliative Care unit, it might not be reasonable to install additional separate digital tools that need to be maintained constantly. In case we cannot introduce the functional prototype in daily procedures, we might give it to the participants in interviews and focus group sessions on collecting feedback on its functionality and for evaluation.

## Data Availability

Not applicable.

## Abbreviations

AI: Artificial Intelligence
HIS: Hospital Information System
MVP: Minimum Viable Product
WMTP: Weekly multi-professional team meeting

## References

[1] Gellerstedt M (2016). The digitalization of health care paves the way for improved quality of life. J System Cybernet Inform.

[2] Frennert S (2019). Lost in digitalization? Municipality employment of welfare technologies. Disabil Rehabil Assist Technol.

[3] Huchler N (2017). Grenzen der Digitalisierung von Arbeit – Die Nicht-Digitalisierbarkeit und Notwendigkeit impliziten Erfahrungswissens und informellen Handelns. Z Arb Wiss.

[4] Cassell EJ (1998). The Nature of Suffering and the Goals of Medicine. Loss Grief Care.

[5] Abraham A, Kutner JS, Beaty B (2006). Suffering at the end of life in the setting of low physical symptom distress. J Palliat Med.

[6] Schneider W, Stadelbacher S. Palliative Care und Hospiz. In: Kriwy P, Jungbauer-Gans M, editors. Handbuch Gesundheitssoziologie. Wiesbaden: Springer Fachmedien Wiesbaden; 2020. p. 481–509. doi:10.1007/978-3-658-06392-4_28.

[7] Finucane AM, O'Donnell H, Lugton J, Gibson-Watt T, Swenson C, Pagliari C (2021). Digital health interventions in palliative care: a systematic meta-review. NPJ Digit Med.

[8] Mills J, Fox J, Damarell R, Tieman J, Yates P (2021). Palliative care providers' use of digital health and perspectives on technological innovation: a national study. BMC Palliat Care.

[9] Professional Record Standards Body. Palliative and End of Life Care. 2022. https://theprsb.org/standards/palliativeandendoflifecare/. Accessed 19 Jan 2023.

[10] Millington-Sanders C, Nadicksbernd JJ, O'Sullivan C, Morgan T, Raleigh A, Yeun P, Ormerod G (2012). Electronic palliative care co-ordination system: an electronic record that supports communication for end-of-life care - a pilot in Richmond, UK. London J Prim Care (Abingdon).

[11] Schnell MW, Hochmuth A, Schulz-Quach C. Palliative Care im Zeitalter der Digitalisierung. In: Schnell MW, Schulz-Quach C, editors. Basiswissen Palliativmedizin. Berlin, Heidelberg: Springer Berlin Heidelberg; 2019. p. 297–302. doi:10.1007/978-3-662-59285-4_20.

[12] Hielscher V, Nock L, Kirchen-Peters S (2015). Technikeinsatz in der Altenpflege: Potenziale und Probleme in empirischer Perspektive.

[13] Beck S (2018). Zum Einsatz von Robotern im Palliativ- und Hospizbereich. MedR.

[14] Bendel O (2018). Pflegeroboter.

[15] Jungtäubl M, Weihrich M, Kuchenbaur M. Digital forcierte Formalisierung und ihre Auswirkungen auf die Interaktionsarbeit in der stationären Krankenpflege: SSOAR - GESIS Leibniz Institute for the Social Sciences, AIS-Studien. 2018;11(2):176–91. 10.21241/ssoar.64872.

[16] May S, Bruch D, Gehlhaar A, Linderkamp F, Stahlhut K, Heinze M (2022). Digital technologies in routine palliative care delivery: an exploratory qualitative study with health care professionals in Germany. BMC Health Serv Res.

[17] Nwosu AC, McGlinchey T, Sanders J, Stanley S, Palfrey J, Lubbers P (2022). Identification of Digital Health Priorities for Palliative Care Research: Modified Delphi Study. JMIR Aging..

[18] Glaser BG, Strauss AL (1967). The Discovery of Grounded Theory: Strategies for Qualitative Research.

[19] System AS, Theory I (2018). Describing Interactions between Work Systems CAIS.

[20] Alter S (2013). Work System Theory: Overview of Core Concepts, Extensions, and Challenges for the Future. JAIS.

[21] Knappertsbusch F, Langfeldt B, Kelle U. Mixed-Methods and Multimethod Research. In: Hollstein B, Greshoff R, Schimank U, Weiß A, editors. Soziologie - Sociology in the German-Speaking World: De Gruyter; 2021. p. 261–272. doi:10.1515/9783110627275-018.

[22] Gimpel H, Kerpedzhiev G, König F, Meindl O. "Teaching an Old Work System New Tricks: Towards an Integrated Method for Work System Transformation in Times of Digitalization". In Proceedings of the 28th European Conference on Information Systems (ECIS), An Online AIS Conference. 2020. https://www.aisel.aisnet.org/ecis2020_rp/6.

[23] Alter S (2006). The work system method: Connecting people, processes, and IT for business results.

[24] Hammersley A (2019). Ethnography: Principles in Practice.

[25] O'Reilly K. Ethnographic Methods: Routledge; 2nd Edition. 2012. 10.4324/9780203864722.

[26] Corbin J, Strauss A (1998). Basics of qualitative research: Techniques and procedures for developing grounded theory.

[27] Breidenstein G, Hirschauer S, Kalthoff H, Nieswand B (2013). Ethnografie: Die Praxis der Feldforschung.

[28] Katz J (2019). On Becoming an Ethnographer. J Contemp Ethnogr.

[29] Bowen GA (2006). Grounded Theory and Sensitizing Concepts. Int J Qual Methods.

[30] Stewart DW (1990). Focus groups: Theory and practice.

[31] Stiel S, Matthes ME, Bertram L, Ostgathe C, Elsner F, Radbruch L (2010). Validierung der neuen fassung des minimalen dokumentationssystems (MIDOS(2)) für patienten in der palliativmedizin : deutsche version der edmonton symptom assessment scale (ESAS) [Validation of the new version of the minimal documentation system (MIDOS) for patients in palliative care : the German version of the edmonton symptom assessment scale (ESAS)]. Schmerz.

[32] Ostgathe C, Wendt KN, Heckel M, Kurkowski S, Klein C, Krause SW (2019). Identifying the need for specialized palliative care in adult cancer patients - development and validation of a screening procedure based on proxy assessment by physicians and filter questions. BMC Cancer.

[33] Devlin J, Chang M-W, Lee K, Toutanova K. BERT: Pre-training of Deep Bidirectional Transformers for Language Understanding. In: Proceedings of the 2019 Conference of the North American Chapter of the Association for Computational Linguistics: Human Language Technologies, Volume 1 (Long and Short Papers). Minneapolis: Association for Computational Linguistics; 2018. p. 4171–86. 10.18653/v1/N19-1423.

[34] Brown T, Mann B, Ryder N, Subbiah M, Kaplan JD, Dhariwal P, et al. Language Models are Few-Shot Learners. In: H. Larochelle, M. Ranzato, R. Hadsell, M.F. Balcan, H. Lin, editors: Curran Associates, Inc; 2020. p. 1877–1901.

[35] Azuaje F, Dubitzky W, Black N, Adamson K (1999). Improving clinical decision support through case-based data fusion. IEEE Trans Biomed Eng.

